# Impact of a low FODMAP diet on the amount of rectal gas and rectal volume during radiotherapy in patients with prostate cancer – a prospective pilot study

**DOI:** 10.1186/s13014-020-1474-y

**Published:** 2020-01-30

**Authors:** Christian Schaefer, Constantinos Zamboglou, Natalja Volegova-Neher, Carmen Martini, Nils Henrik Nicolay, Nina-Sophie Schmidt-Hegemann, Paul Rogowski, Minglun Li, Claus Belka, Arndt-Christian Müller, Anca-Ligia Grosu, Thomas Brunner

**Affiliations:** 1 Department of Radiation Oncology, University Hospital, LMU, Marchioninistr. 15, 81377 Munich, Germany; 2 Department of Radiation Oncology, Medical Center, University of Freiburg, Faculty of Medicine, Freiburg, Germany; 3 German Cancer Consortium (DKTK), Partner Site Freiburg, Freiburg, Germany; 4 German Cancer Consortium (DKTK), Partner Site Munich, Munich, Germany; 5 University Clinic for Radiation Oncology, University Hospital Tübingen, Tübingen, Germany; 6 German Cancer Consortium (DKTK), Partner Site Tübingen, Tübingen, Germany; 7 University Clinic for Radiation Therapy, University Hospital Magdeburg, Magdeburg, Germany

**Keywords:** Radiotherapy - prostate Cancer, Low FODMAP diet, Rectal volume, Rectal gas

## Abstract

**Background:**

Small inter- and intrafractional prostate motion was shown to be a prerequisite for precise radiotherapy (RT) of prostate cancer (PCa) to achieve good local control and low rectal toxicity. As rectal gas and rectal volume are known to have a relevant effect on prostate motion, this study aims to reduce these parameters by using a Low FODMAP Diet (LFD) and to show feasibility of this intervention.

**Methods:**

We compared a prospective intervention group (IG, *n* = 25) which underwent RT for PCa and whose patients were asked to follow a LFD during RT with a retrospective control group (CG, *n* = 25) which did not get any dietary advice. In the planning CT scan and all available cone beam CT scans rectal gas was classified based on a semiquantitative score (scale from 1 to 5) and rectal volume was measured. Furthermore, patients’ compliance was evaluated by a self-assessment questionnaire.

**Results:**

Clinical and treatment characteristics were well balanced between both groups. A total of 266 (CG, 10.6 per patient) and 280 CT scans (IG, 11.2 per patient), respectively, were analysed. The frequency distribution of gas scores differed significantly from each other (*p* < .001) with the IG having lower scores. Rectal volume was smaller in the IG (64.28 cm^3^, 95% CI 60.92–67.65 cm^3^, SD 28.64 cm^3^) than in the CG (71.40 cm^3^, 95% CI 66.47–76.32 cm^3^, SD 40.80 cm^3^) (*p* = .02). Mean intrapatient standard deviation as a measure for the variability of rectal volume was 22 cm^3^ in the IG and 23 cm^3^ in the CG (*p* = .81). Patients’ compliance and contentment were satisfying.

**Conclusions:**

The use of a LFD significantly decreased rectal gas and rectal volume. LFD was feasible with an excellent patients’ compliance. However, prospective trials with a larger number of patients and a standardized evaluation of gastrointestinal toxicity and quality of life are reasonable.

**Trial registration:**

German Clinical Trials Register, DRKS00012955. Registered 29 August 2017 - Retrospectively registered, https://www.drks.de/drks_web/navigate.do?navigationId=trial.HTML&TRIAL_ID=DRKS00012955

## Background

**Fig. 1:**
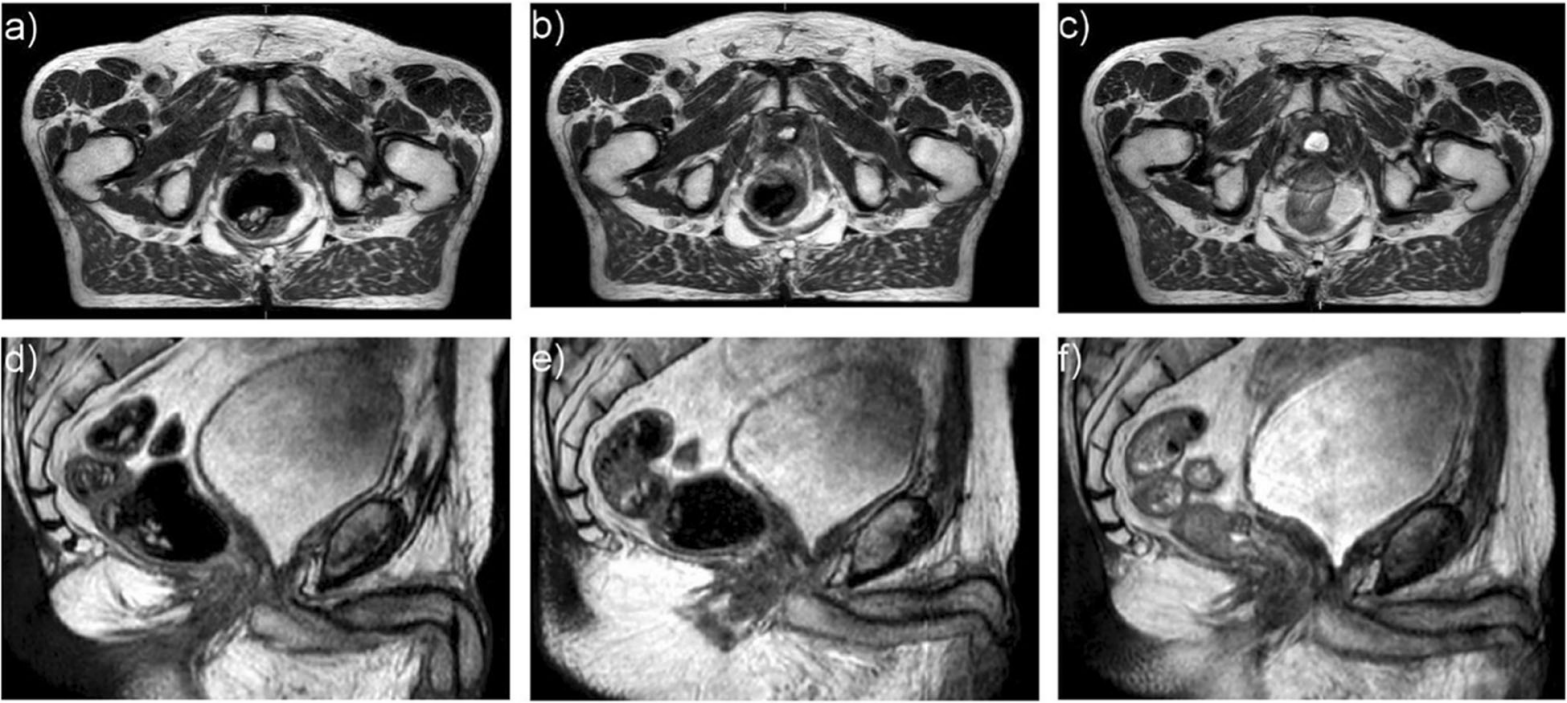
T2-weighted MR sequence of a postprostatectomy patient during MR-guided salvage radiotherapy at MR Unity (Elekta®). Axial (**a-c**) and the corresponding sagittal (**d-f**) views demonstrate different rectal volumes and different amounts of rectal gas during the course of fractionated radiotherapy

In Germany, prostate cancer (PCa) is the most common male cancer and it ranks third among the causes of cancer death in men [1]. Radiotherapy (RT) plays an important role in its treatment, both as a definitive and as a postoperative treatment option, e.g. in patients with persisting PSA values after prostatectomy or with a biochemical or local recurrence. Effectiveness of RT increases with the administered dose to the prostate [2, 3]. However, toxicity also increases with the administered dose to the surrounding normal tissue. Thus, RT should be as exact as possible. One problem in achieving an exact RT is the positional variability of the prostate which can be divided into interfractional motion (organ motion between the fractions) and intrafractional motion (organ motion during a fraction). Prostate motion seems to be influenced relevantly by bladder filling and particularly by rectal filling [4–6]. Figure 1 shows an example for differences in rectal filling. An increased rectal volume and an increased amount of mobile rectal gas correlates with rectal movements and intrafractional motion [7, 8]. Furthermore, a large rectal volume in the planning CT scan leads to an increased interfractional motion and consequently to a reduced local and biochemical tumour control as well as to increased gastrointestinal toxicity [9]. In recent years, non-inferiority of hypofractionated RT of PCa could be shown [10], and even ultra-hypofractionated regimens (stereotactic body radiotherapy, SBRT) were shown to be non-inferior to normofractionated RT [11]. Considering the increasing use of these new techniques with their longer time and higher dose per radiation fraction, prostate motion becomes even more important. Different approaches have been proposed to reduce prostate motion by reducing the extent and the variability of rectal volume or the amount of rectal gas during RT, including mechanical approaches like the daily application of endorectal balloons or daily enema. While the effectiveness of the endorectal balloon remains unclear, the use of daily enema seems to have a positive impact on both intra- and interfractional motion [12–14]. Thus, in some radiooncology centers it is part of the clinical routine [15]. However, considering the invasive nature of these methods there still is a need for an effective and less invasive approach to reduce organ motion. Since the daily intake of a laxative, magnesium oxide, was unsuccessful [16, 17], the focus has shifted to dietary interventions. A number of trials already have evaluated dietary interventions during RT in patients with PCa to reduce rectal volume, rectal gas, prostate motion or gastrointestinal toxicity [14, 18–24]. In the majority of these trials patients were asked to follow antiflatulent diets or they were instructed regarding their intake of fibres and fluids, partly in combination with laxatives. Nevertheless, despite some promising results most of these trials did not observe significant effects (see discussion for details) and none of these approaches have been implemented on a large scale in clinical routine.

In the current trial we evaluated for the first time a Low Fodmap Diet (LFD) which is usually used in patients with irritable bowel syndrome or chronic inflammatory bowel diseases. The acronym FODMAP stands for “fermentable oligo-, di- and monosaccharides and polyols”. FODMAPS are short-chain carbohydrates characterized by poor absorbability, high fermentability and high osmotic potential. These characteristics lead to a large quantity of substrates in the gut, a high water influx into the intestinal lumen and a high gas production in the colon [25, 26]. Consequently, we hypothesized that patients who follow a LFD during RT of PCa have a reduced amount of rectal gas and a smaller and less variable rectal volume compared to patients who eat normally.

## Methods

The current trial was a controlled pilot study with 25 patients in each arm. A retrospective control group (CG), which did not get any dietary advice was compared to a prospective intervention group (IG), which was asked to follow a LFD during RT.

All patients were treated at the department for radiation oncology of the University Hospital Freiburg in the period from August 2014 to October 2016 (CG) and from November 2016 to January 2018 (IG), respectively. Eligible patients had to be 18 years or older, have PCa and receive a definitive, an adjuvant or a salvage RT. Patients in the IG had to sign an informed consent. Patients could not be included if they had prior rectal surgery or chronic inflammatory bowel disease, if they regularly took opioids or if RT included the pelvic lymphatic pathways. Following approval by the institutional ethics committee and registration in the German Clinical Trials Register (DRKS00012955), patients in the IG were enrolled within the frame of their first visit of the department of radiation oncology. Patients in the CG were randomly chosen by a physician who was not involved in the evaluation.

### Radiotherapy

All patients were treated according to the standard operation procedure of the Department of Radiation Oncology of the University Hospital Freiburg. In the definitive setting patients received a normofractionated intensity-modulated RT (IMRT) with single doses of 2 Gy and a total dose of 74 to 78 Gy. Adjuvant RT was performed in case of R1-resection, T3-status or a Gleason score ≥ 7b. In the postoperative (adjuvant or salvage) setting a normofractionated (1.8 Gy per fraction) 3D conformal RT to a dose of 14.4 to 19.8 Gy was used, followed by IMRT to a cumulative total dose of 66.6 Gy. In case of a R1-resection or a Gleason score ≥ 8 the total dose of adjuvant RT was escalated to 70.2 Gy. Moreover, in case of a PET-positive local recurrence a boost to the PET-positive tumour to a total dose of 70.2 or 72 Gy was used [27]. For image-guidance, cone beam CTs were acquired before the first three fractions and after that at least weekly. Patients were asked to empty their rectum before every RT fraction and to drink 750 ml of water 60 min before every RT fraction. In both groups there were no restrictions regarding the use of supportive care drugs such as macrogol in case of obstipation and simeticon in case of flatulence.

### Intervention

Patients of the IG were informed about the trial at their first visit to the outpatient clinic and received a leaflet containing information about the trial and the LFD. Furthermore, they received a list with food to be avoided because of its high FODMAP concentration and possible alternative food products with a low FODMAP concentration (see Additional file 1:). The list was based on lists which had already been published [28, 29] and was divided into the categories “dairy products”, “grain products”, “fruit”, “vegetables”, “nuts”, “legumes”, “honey / syrup”, “sweeteners” and “beverages” for an easy handling. Patients were asked to start with the LFD immediately after their presentation to the outpatient clinic and to follow the diet until the last day of RT. Moreover, they were asked to self-assess their adherence to the diet every day using a simple questionnaire.

### Data collection

Patient data and tumour data were extracted from the electronic patient file. For contouring, Nucletron Oncentra Treatment Planning System 4.3 was used. The planning CT scan and all available cone beam CT scans were loaded and coregistered by using a mutual information registration with a clipbox. Of 560 loaded CT scans, 546 were suitable for the data collection while 14 scans could not be used due to a too small field of view or strong artefacts by hip prostheses. In both groups this corresponds to a mean number of 11 CT scans per patient (see Table 1).

**Table 1:** Patients’ and treatment characteristics

	KG (*n* = 25)	IG (*n* = 25)	statistics
Indication			*p* = *.62* ^1^
primary RT	6 (24%)	6 (24%)	
adjuvant RT	10 (40%)	7 (28%)	
salvage RT	9 (36%)	12 (48%)	
T-stage			*p = 1.00* ^*2*^
T2	12 (48%)	11 (44%)	
T2a	4 (16%)	1 (4%)	
T2b	1 (4%)	0	
T2c	6 (24%)	10 (40%)	
T2 (unspecified)	1 (4%)	0	
T3	13 (52%)	13 (52%)	
T3a	12 (48%)	10 (40%)	
T3b	1 (4%)	2 (8%)	
T3 (unspecified)	0	1 (4%)	
T4	0	1 (4%)	
N-stage			*p = 1.00* ^*2*^
N0	22 (88%)	23 (92%)	
N1	3 (12%)	2 (8%)	
M-stage			*p = 1.00* ^*2*^
M0	24 (96%)	24 (96%)	
M1	1 (4%)	1 (4%)	
Gleason-Score			*p* = *.22* ^*2*^
6	2 (8%)	1 (4%)	
7a	10 (40%)	6 (24%)	
7b	6 (24%)	14 (56%)	
8	4 (16%)	3 (12%)	
9	3 (12%)	1 (4%)	
D’Amico risk			*p = .50* ^*2*^
low risk	2 (8%)	0	
intermediate risk	4 (16%)	6 (24%)	
high risk	19 (76%)	19 (76%)	
ADT			*p = 1.00* ^*2*^
yes	4 (16%)	3 (12%)	
no	21 (84%)	22 (88%)	
Age in years			*p = .09* ^*3*^
Mean (SD)	69.3 (6,5)	65.9 (7,2)	
PSA in ng/ml			
primary RT: Mean (SD)	16.3 (18.9)	5.96 (5.9)	*p = .39* ^*4*^
adjuvant / Salvage RT: Mean (SD)	0.3 (0.4)	0.7 (1.2)	*p = .31* ^*4*^
Total Dose in Gy			
primary RT: Mean (SD)	73.9 (2.6)	76 (1.8)	*p = .14* ^*3*^
adjuvant / Salvage RT: Mean (SD)	68.7 (2.2)	69.1 (2.1)	*p = .71* ^*4*^
Usable CT scans			
Mean (SD)	10.6 (2.0)	11.2 (2.6)	*p = .19* ^*4*^

*1: chi square test, 2: Fisher’s exact test, 3: T-test, 4: Mann-Whitney-U-test*

The rectal volume was calculated by contouring the external surface of the rectum in every slice (2 mm) from the nearest slice to the lower edge of the fourth sacral vertebra until the nearest slice to 2 cm cranial of the anus.

The amount of rectal gas was measured by using a five-tier semiquantitative score described by McNair et al. [21]. For evaluation, the sagittal CT slices were screened and the amount of gas was estimated and categorized (see Table 2) using the same cranial and caudal borders which have already been used for measuring the rectal volume were used.

**Table 2:** Semiquantitative gas score by McNair et al. [21] and its frequency distribution

Score	Gas‘proportion of rectal volume	CG (*n* = 266)	IG (*n* = 280)
1	0 – 5%	16 (6.0%)	24 (8.6%)
2	5 – 25%	94 (35.3%)	131 (46.8%)
3	25 – 50%	73 (27.4%)	82 (29.3%)
4	50 – 75%	67 (25.2%)	35 (12.5%)
5	75 – 100%	16 (6.0%)	8 (2.9%)

### Statistical analyses

Statistical analyses were performed with SPSS 22 and SPSS 25 (IBM). Primary endpoint was the amount of rectal gas. For comparing the groups, all CT scans were considered, in analogy to previous trials [16, 18]. Keeping in mind the ordinal scaled data, analysis was performed with the Mann-Whitney-U-Test. Furthermore, in analogy to McNair et al. the proportion of CT scans with a score of “4” or “5” for every patient was determined [21], and groups were compared using the Mann-Whitney-U-Test. Secondary endpoints were the rectal volume and its variability. Regarding rectal volume, groups were again compared based on all CT scans using students’ t-test. In analogy to Oates et al. the standard deviation of every patient was calculated to compare the intrapatient variability of rectal volume [24]. Analysis was performed with the Mann-Whitney-U-Test. Finally, patients’ compliance in the IG is reported in a descriptive way.

## Results

Both groups did not differ significantly in any of the patients’ or treatment characteristics (see Table 1) and thus can be well compared. No patient of the IG disrupted the LFD prematurely, so all patients and all appropriate CT scans could be used for analyses. In total, 280 CT scans in the IG (11.2 per patient) and 266 CT scans in the CG (10.6 per patient), respectively, were analysed (*p* = .19).

### Rectal gas amount

**Fig. 2:**
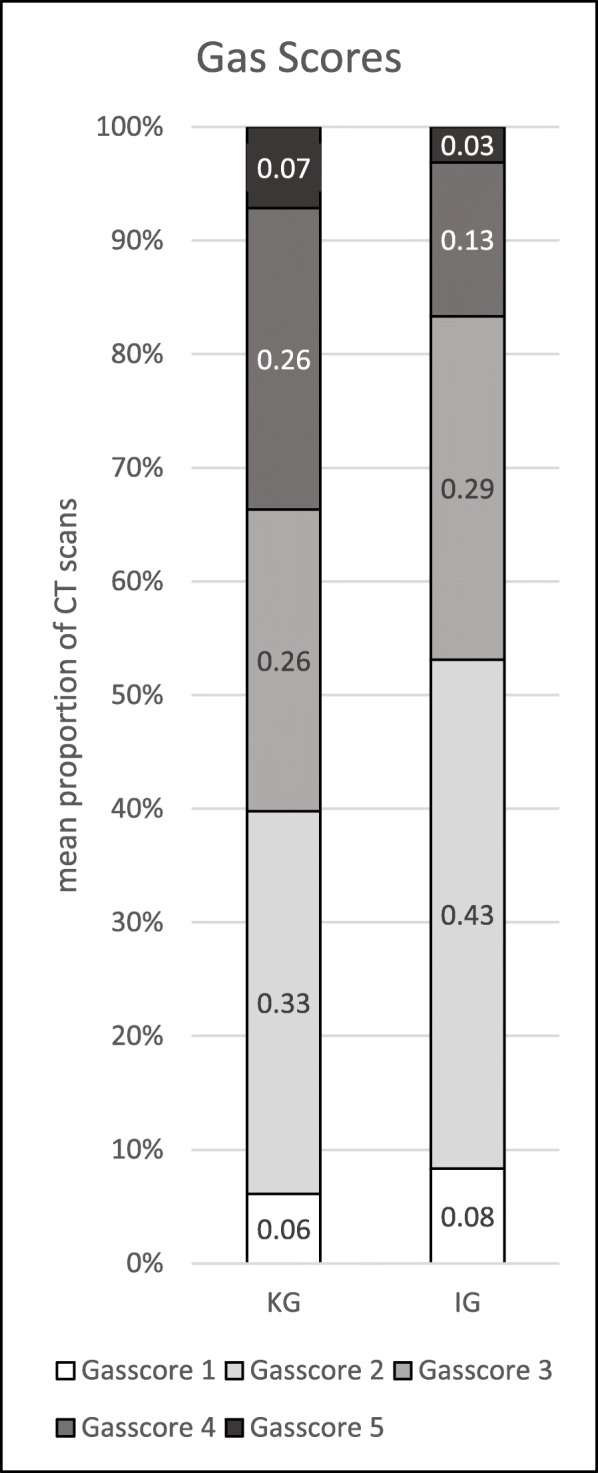
Mean proportion of gas scores

The frequency distribution of gas scores in both groups differed highly significantly from each other (*p* < .001) with the IG having lower gas scores as is shown in Table 2. The mean proportion of CT scans with a gas score of “4” or “5” was 16% in the IG (95 CI 0.10–0.22, SD 0.14) and 33% in the CG (95% CI 0.21–0.44, SD 0.27) and differed significantly from each other (*p* = .028). Mean proportions of CT scans with each gas score are shown in Fig. 2.

### Rectal volume

**Fig. 3:**
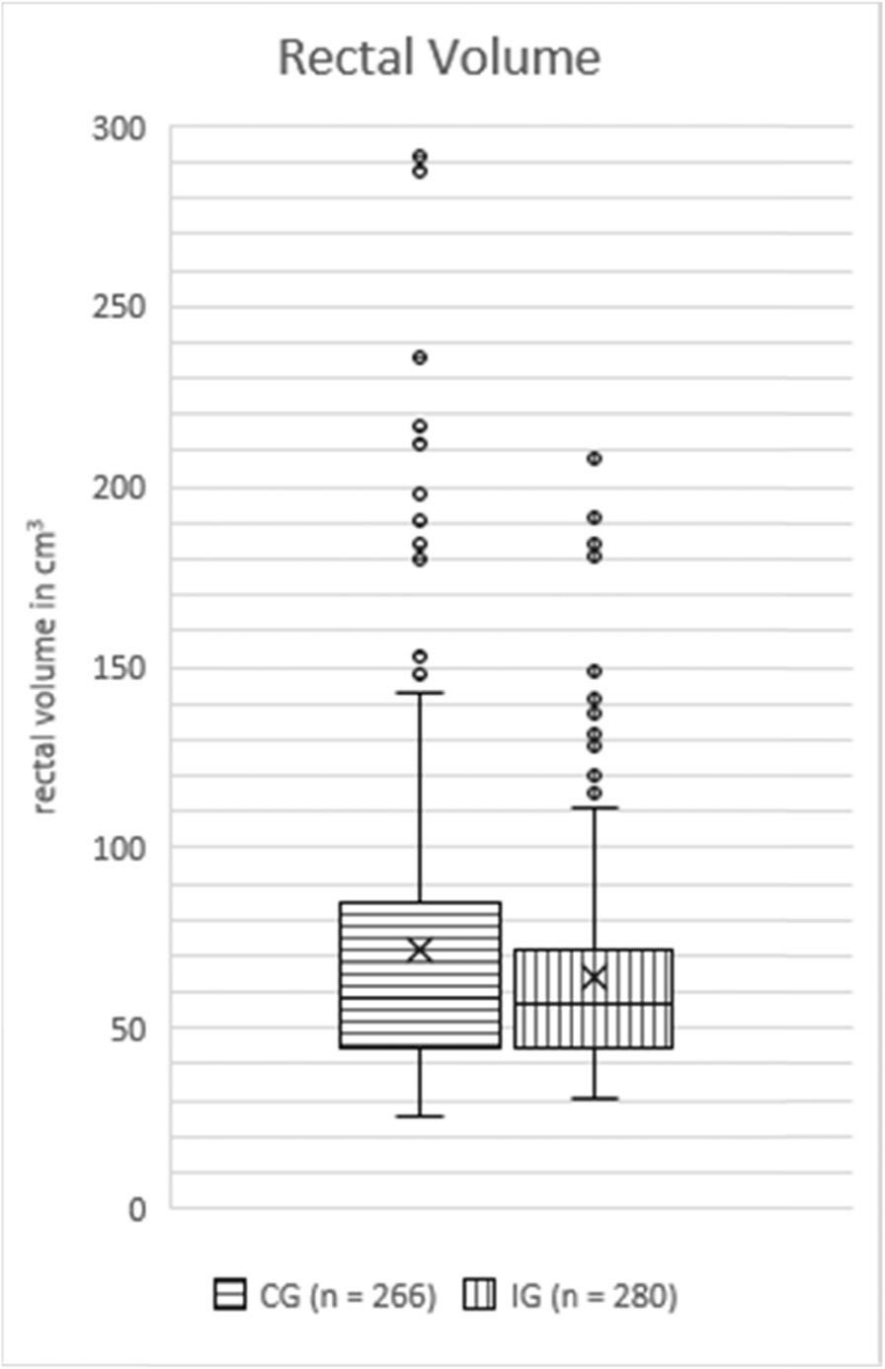
Boxplot of rectal volume

Mean rectal volume was 64.28 cm3 (95% CI 60.92–67.65 cm3, SD 28.64 cm3) in the IG and 71.40 cm3 (95% CI 66.47–76.32 cm3, SD 40.80 cm3) in the CG and differed significantly from each other (*p* = 0.02, see Fig. 3).

### Rectal volume variability

**Fig. 4:**
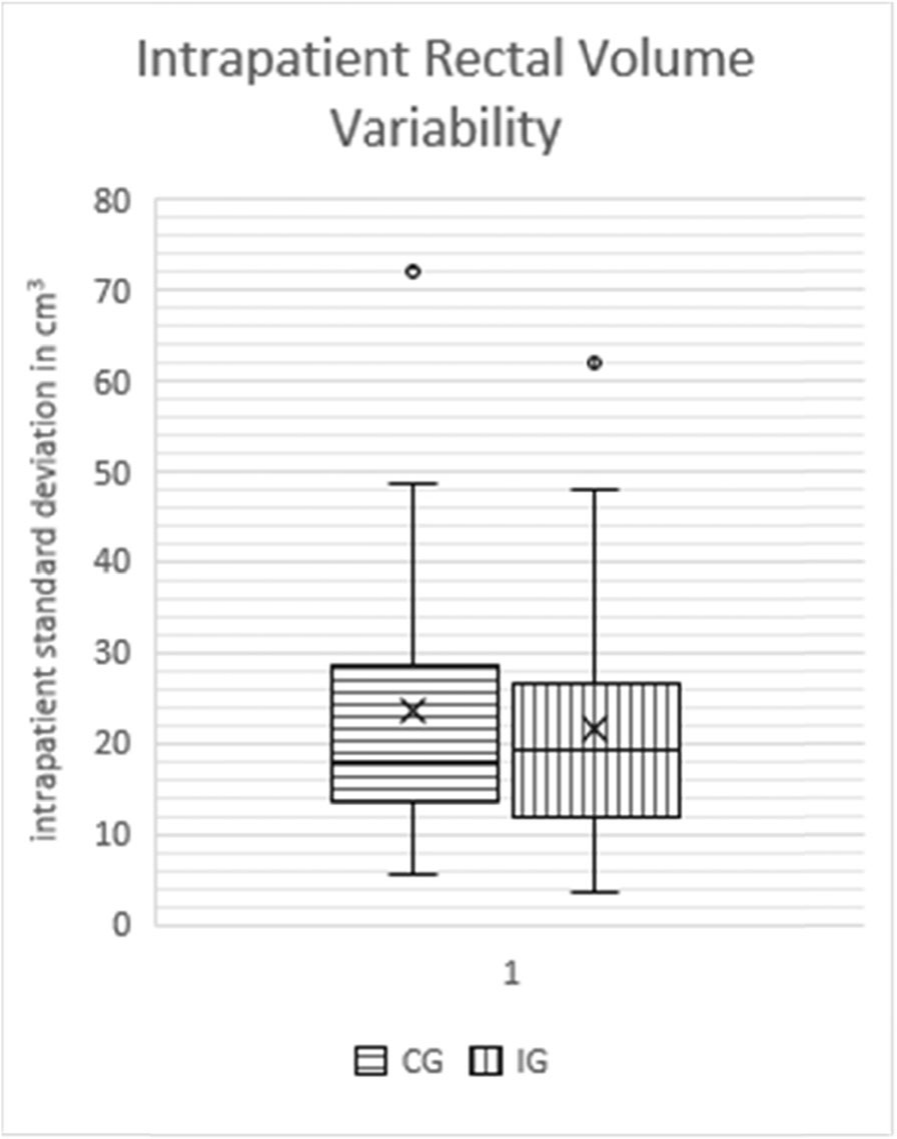
Boxplot of intrapatient rectal volume variability

Mean intrapatient standard deviation was 21.52 cm3 in the IG (95% CI 15.86–27.18 cm3, SD 13.71 cm3) and 23.44 cm3 in the CG (95% CI 16.87–30.01 cm3, SD 15.91 cm3) and did not differ significantly from each other (*p* = 0.81, see Fig. 4).

### Compliance

Twenty-four of 25 patients in the IG returned completely filled self-assessment questionnaires. Mean duration of the LFD and the self-assessment was 59 days (SD 6.01). Patients fulfilled the diet guidelines at 49% (95 CI 0.39–0.59, SD 0.24) of the days “completely”, at 39% (95 CI 0.31–0.47, SD 0.19) “pretty much”, at 8% (95 CI 0.05–0.11, SD 0.08) “more or less”, at 5% (95 CI 0.02–0.07, SD 0.06) “rather not” and at 0% (95 CI 0.00–0.01, SD 0.01) “not at all”. Moreover, at the end of RT 20 of 21 patients agreed with the statement “I would recommend the LFD to a friend in the same situation” and 13 of 21 patients agreed with the statement “I could imagine following a LFD regardless of the RT”. Regarding their satisfaction with digestion, 11 of 21 patients (52%) were more content than before the treatment, 6 (29%) reported the same degree of contentment and 4 (19%) were less content.

## Discussion

In the current pilot study, following a LFD during local RT of PCa led to a reduced amount of rectal gas and to a reduced rectal volume. Intrapatient variability of rectal volume did not change significantly. Overall, patients’ compliance was good.

The amount of rectal gas was significantly reduced by following a LFD during RT of PCa. This corresponds with previous data showing that intake of FODMAPs leads to an increased breath hydrogen level and to a higher amount of gas in the colon assessed by magnetic resonance imaging (MRI) [30, 31]. Taking into account that hypofractionated and ultra-hypofractionated concepts are more and more frequently employed, a reduction of intrafractional motion will be even more important in future. Thus, the result of the current pilot study is very promising as rectal gas is responsible for a relevant part of intrafractional prostate motion [7].

Rectal volume was siginificantly reduced by following a LFD as well. This also is in line with previous data which showed a reduction of stool volume and weight by a LFD in ileostomates [32]. Moreover, a MRI study showed that FODMAPs lead to a higher small bowel water content, to more colonic gas and to larger diameters of the small and large bowel with the impact depending on the subgroup of FODMAPs [31]. Hence, it will probably be helpful for future trials to distinguish further between different FODMAP subgroups in the study design. Furthermore, increasing interest in the LFD over the last years led to more extensive food lists declaring FODMAP concentrations. Thus, even better dietary advice can be given for future studies. It was observed that the full symptom-relieving effect of the LFD occurred after 7 days in patients with irritable bowel syndrome [33]. Assuming that achieving the full effect on rectal gas and volume needs a similar period of time, patients in the current trial started too late with the LFD (median 2.5 days before start of RT). There was probably just a partial effect at the time of the planning CT scan and the first few cone beam CT scans. Considering these aspects, the reduction of rectal volume in this trial already seems very encouraging.

Intrapatient variability of the rectal volume was not reduced by a LFD. To our knowledge there is no previous study evaluating this specific question. Keeping in mind the mobility of rectal gas and the reduction of rectal gas and rectal volume in the current study, one would expect a less variable rectal volume. Thus, regarding this, an evaluation with a higher sample size would be of interest. Moreover, there may be even better measures for this question than the standard deviation, such as the proportion of scans with values outside a certain range, e.g. the interquartile range.

Furthermore, we collected compliance data in the IG by using a self-assessment questionnaire. At 88% of the days patients adhered to the dietary advice “completely” or “pretty much”, which in our opinion is a satisfying result. In previous studies a similarly good compliance was observed as well [33, 34]. Patients reported that the LFD was easy to implement and that the taste was good. However, LFD was slightly more expensive (10% compared to a standard diet) and approximately one third of patients had to go to special stores to buy their food. Compliance was reduced by living together with family members or flatmates [35]. An important role in achieving the satisfying compliance probably played the high degree of satisfaction with the digestion compared to the time before start of the treatment. Furthermore, from the psychological point of view it may have had a positive impact on compliance that patients in the IG felt able to contribute to a successful treatment. Moreover, lists showing the FODMAP concentration of different food products constantly get more and more complete. Thus, it will be even easier for patients to reduce their FODMAP intake in future, which will probably improve compliance further.

First of all, the study was not randomised and not blinded due to the study design with a retrospective CG and a prospective IG. However, the design also had a positive effect as patients of the IG were not able to talk to patients of the CG and to influence their diet. On the other side, due to the study design patients with heterogenous RT concepts regarding applied doses to the target regions and RT techniques were included. Thus, despite well comparable groups regarding RT indications (see Table 1) there may have been differences in rectal doses between the groups regardless of the diet intervention. This is the reason why we did not assess any acute or chronic gastrointestinal toxicity in this study which is surely the most relevant endpoint. Further studies should evaluate also the effect of a LFD on acute and chronic gastrointestinal toxicity, which requires homogenous groups regarding dose, target volumes, RT techniques and secondary illnesses and a prospective setting with standardised methods for toxicity assessment. Second, the use of a semiquantitative score in combination with the missing blinding potentially could have led to a confirmation bias. Third, analogous to previous studies [16, 18] in this trial all CT scans (*n* = 546) were considered for analysis of rectal gas and volume. This seems reasonable as every outlier is clinically very relevant, especially during hypo- or ultrahypofractionated RT. The difference in rectal volume did not reach statistical significance when comparing patients’ mean rectal volumes between the groups (*p* = 0.75): 64.48 cm^3^ in the IG (95% CI 57.63–71.34, SD 16.62 cm^3^) vs. 74.62 cm^3^ in the CG (95% CI 58.55–90.69, SD 38.94 cm^3^). Based on the data gathered in the current study, a sample size of 196 patients would be needed to evaluate the effect of a LFD on patients’ mean rectal volume (calculated with G-Power 3.1.9.2 with α = 0.05 and 1- β= 0.8). The frequency distribution of patients’ median gas scores in both groups differed significantly from each other as well (*p* = .049). Finally, also inflammation in the bowel may influence the rectal gas amount which was the reason for the exclusion of patients with known chronic inflammatory bowel disease. However, acute inflammatory processes in the rectum due to RT were not assessed in our study and likewise their effect on rectal gas volume is unclear.

A strength of the current study is first of all the presence of a control group whose patients could not be influenced in their diet by the intervention group due to the study design. Other positive aspects are the high number of analysed CT scans and the evaluation of patients’ compliance.

Other trials evaluating dietary interventions to reduce rectal volume, rectal gas, prostate motion or gastrointestinal side effects during RT of PCa were published: Smitmans et al. (2008) tested an antiflatulent diet in combination with magnesium oxide and found a significant reduction of feces, gas and moving gas. Moreover, the success rate of 3D-grey value registration was improved significantly and there was a trend to reduced interfractional prostate motion [18]. Nichol et al. (2010) used a one-group design and cine-MRI to also evaluate an antiflatulent diet and magnesium oxide. They could not show a significant decrease in intrafractional prostate motion or the sagittal rectal area [19]. Lips et al. (2011) who also used an antiflatulent diet with magnesium oxide found a significantly increased intrafractional motion when looking at the portal images of all beams [20]. McNair et al. (2011) also used a one-group design and advised patients individually regarding their daily intake of fluids and fibres. This did not lead to a significant reduction of rectal volume. A change in rectal gas scores was the only variable correlating with rectal volume changes [21]. Pettersson et al. (2012 and 2014) asked patients to reduce their intake of insoluble fibres and lactose. There was no significant change in acute or long-term gastrointestinal side effects or other aspects of health-related quality of life [22, 23]. Yahya et al. (2013) used a three-group design and tested a daily microenema and a dietary intervention increasing the intake of fibres and fluids. The study showed a reduced rectal cross-sectional area and fewer geometric misses in the microenema group compared to the control group, but there was no significant effect of the diet [14]. Oates et al. (2013) evaluated an antiflatulent diet in combination with psyllium and found a trend to reduced rectal volume variability [24]. To conclude, these trials mostly did not observe significant effects and their dietary interventions have not been implemented in large scale in clinical routine.

During the time of the recruitment of the current trial a study was published evaluating the impact of the LFD in patients with gynaecological tumours receiving a RT. Compared to the control group, a better quality of life, a lower worsening of performances status during RT and a good compliance could be observed. Gastrointestinal symptoms did not change significantly [36]. Furthermore, a pilot study was published evaluating the impact of the LFD on patients with a chronic radiation enteropathy. The LFD reduced gastrointestinal symptoms significantly and led to a better quality of life [37].

To conclude, the current pilot study showed a significant reduction of rectal gas and rectal volume by the LFD. There was no difference in intrapatient rectal volume variability. Following a LFD during RT of PCa appears to be easily feasible. Hence, considering these promising results, a further prospective randomised trial with a larger sample size seems reasonable. Moreover, a further prospective evaluation should also include a standardised evaluation of gastrointestinal toxicity and patient-reported quality of life.

## Supplementary information

**Additional file 1.** This list shows the nutrition advice for the dietary intervention

## Data Availability

The dataset of the current is are available from the corresponding author on reasonable request.

## Abbreviations

3D-CRT: Three dimensional conformal radiotherapy
CG: Control group
CT: Computer tomography
FODMAP: Fermentable oligo-, di or monosaccharides and polyols
IG: Intervention group
IMRT: Intensity modulated radiotherapy
LFD: Low FODMAP diet
PCa: Prostate cancer
PET: Positron emission tomography, RT radiotherapy
PSA: Prostate specific antigen
SBRT: Stereotactic body radiation therapy
