# Supplementary material for: Impact of a low FODMAP diet on the amount of rectal gas and rectal volume during radiotherapy in patients with prostate cancer – a prospective pilot study
Source: Radiat Oncol. 2020 Jan 30;15:27. doi: 10.1186/s13014-020-1474-y (PMC6993432; doi:10.1186/s13014-020-1474-y)
Supplement: Supplementary file 1 — Additional file 1. This list shows the nutrition advice for the dietary intervention [file 13014_2020_1474_MOESM1_ESM.pdf]

	**food which should be avoided**	**good alternatives**
**dairy products**	cream<br>yogurt<br>cheese *(cream and soft cheese, e.g. ricotta, cottage cheese,…)*<br>milk (cow’s, goat’s and sheep’s milk)<br>pudding<br>quark<br>ice cream	butter<br>yogurt (lactose-free)<br>cheese (hard cheese*, e.g. emmentaler, parmesan,...)*<br>milk (lactose-free)<br>rice milk<br>sorbet
**grain products**	wheat / rye (in large amounts, e.g. bread, pasta, couscous, biscuits)	spelt<br>gluten-free products<br>rice
**fruits**	apples<br>apricots<br>pears<br>canned fruits<br>khakis<br>cherries<br>lychees<br>mangos<br>nectarines<br>peaches<br>plums (also dried)<br>watermelons<br><br>*In general, no large amounts of fruits, dried fruits and fruit juice!*	bananas<br>strawberries<br>grapefruits<br>blueberries<br>raspberries<br>kiwis<br>limes<br>mandarins<br>passion fruits<br>melons (honeydew, cantaloupe)<br>oranges<br>papayas<br>star fruits<br>grapes<br>lemons
**vegetables**	artichokes<br>avocados<br>cauliflower<br>broccoli<br>peas<br>fennel<br>garlic<br>leek<br>mushrooms<br>Brussels sprouts<br>beetroot<br>shallots<br>asparagus<br>cabbage<br>savoy<br>sugar snaps<br>onions	eggplants<br>bamboo shoots<br>beans (green)<br>spring onions (the green part)<br>cucumber<br>carrots<br>potatoes<br>herbs (fresh)<br>pumpkin<br>corn<br>chard<br>pepper<br>parsnips<br>salad<br>chive<br>celery<br>sprout<br>tomatoes<br>zucchini
**legumes**	beans (white)<br>chickpeas<br>kidney beans<br>lentils	*alternative sources of protein:*<br>*e.g. meat, fish, poultry, tofu,…*
**nuts**	cashews<br>pistachios	pumpkin seeds<br>almonds (<10 pieces)
**honey / syrup**	honey	maple syrup<br>sugar syrup
**sweeteners**	fructose<br>isomalt<br>corn syrup<br>maltitol<br>mannitol<br>sorbitol<br>xylitol	acesulfame<br>aspartame<br>glucose<br>dextrose<br>sugar<br><br>*In general, artificial sweeteners which don´t end with „-ol“!*
**beverages**	*all carbonated drinks* (e.g. mineralwater, beer, sparkling wine, lemonade,...)	*all non-carbonated drinks*
